# Extramedullary Acute Myeloid Leukemia Tumor Presenting in the Radial Nerve

**DOI:** 10.5334/jbsr.2788

**Published:** 2022-04-27

**Authors:** Nico Hustings, Veerle Goosens, Geert Vanderschueren

**Affiliations:** 1 UZ Leuven, BE

**Keywords:** Acute, Myeloid, Leukemia, Extramedullary, tumor, radial, nerve, MRI

## Abstract

**Teaching Point:** Extramedullary acute myeloid leukemia tumor belongs to the differential diagnosis when a tumor develops in a patient with a history of leukemia, and magnetic resonance imaging is of diagnostic value by demonstrating iso-intensity and hyperintensity compared to skeletal muscle respectively on T1- and T2-weighted images and homogeneous contrast enhancement.

## Case History

**Figure 1:**
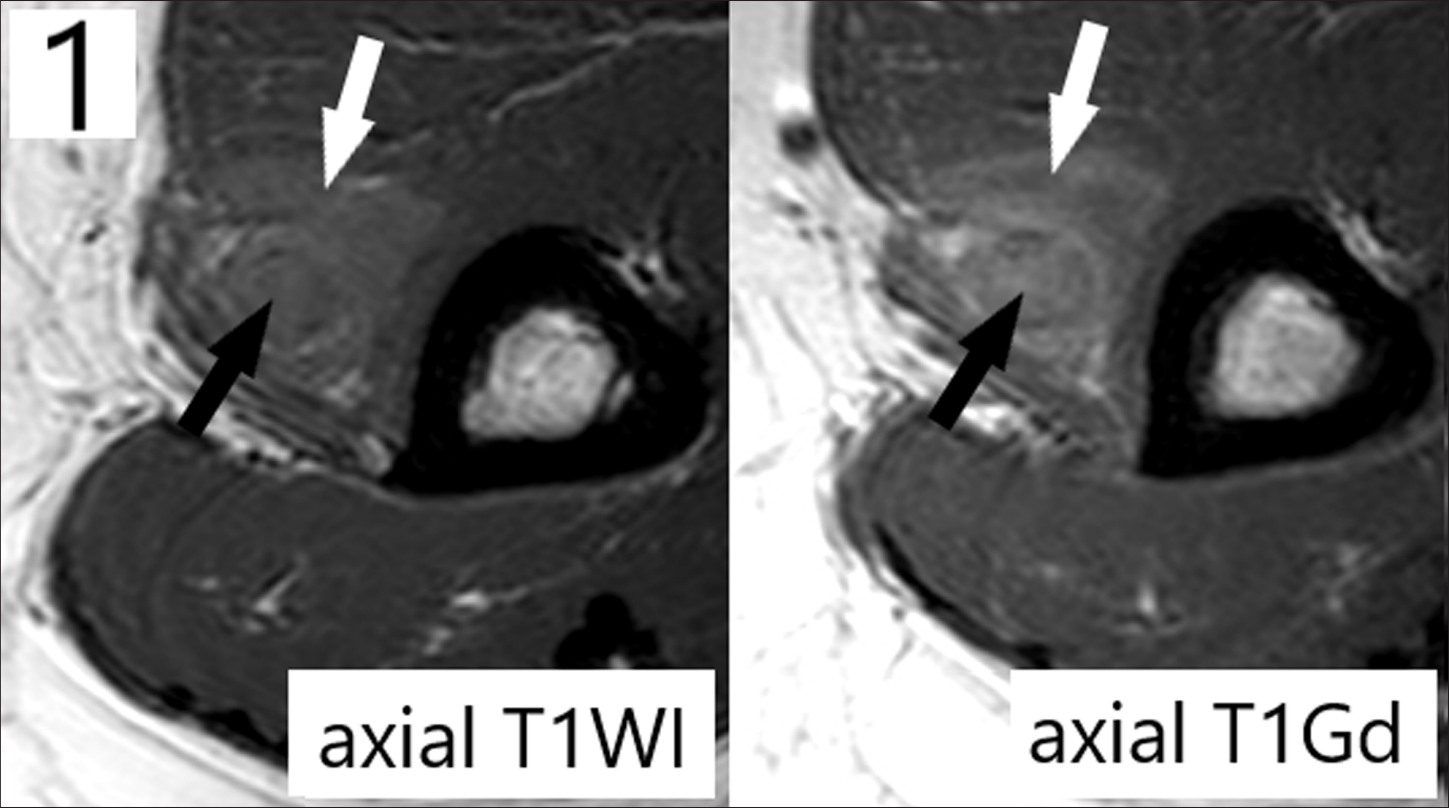


**Figure 2:**
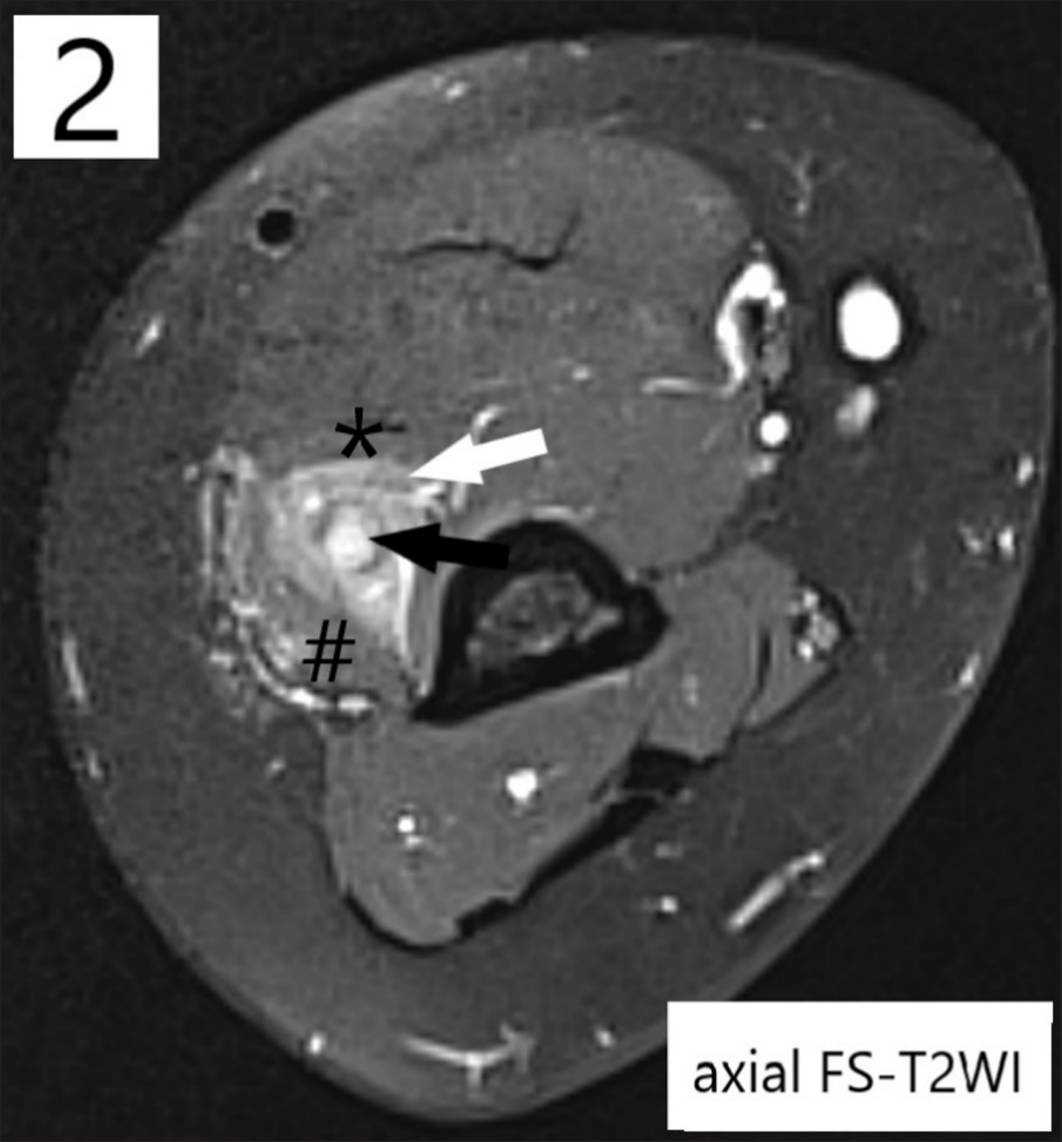


**Figure 3:**
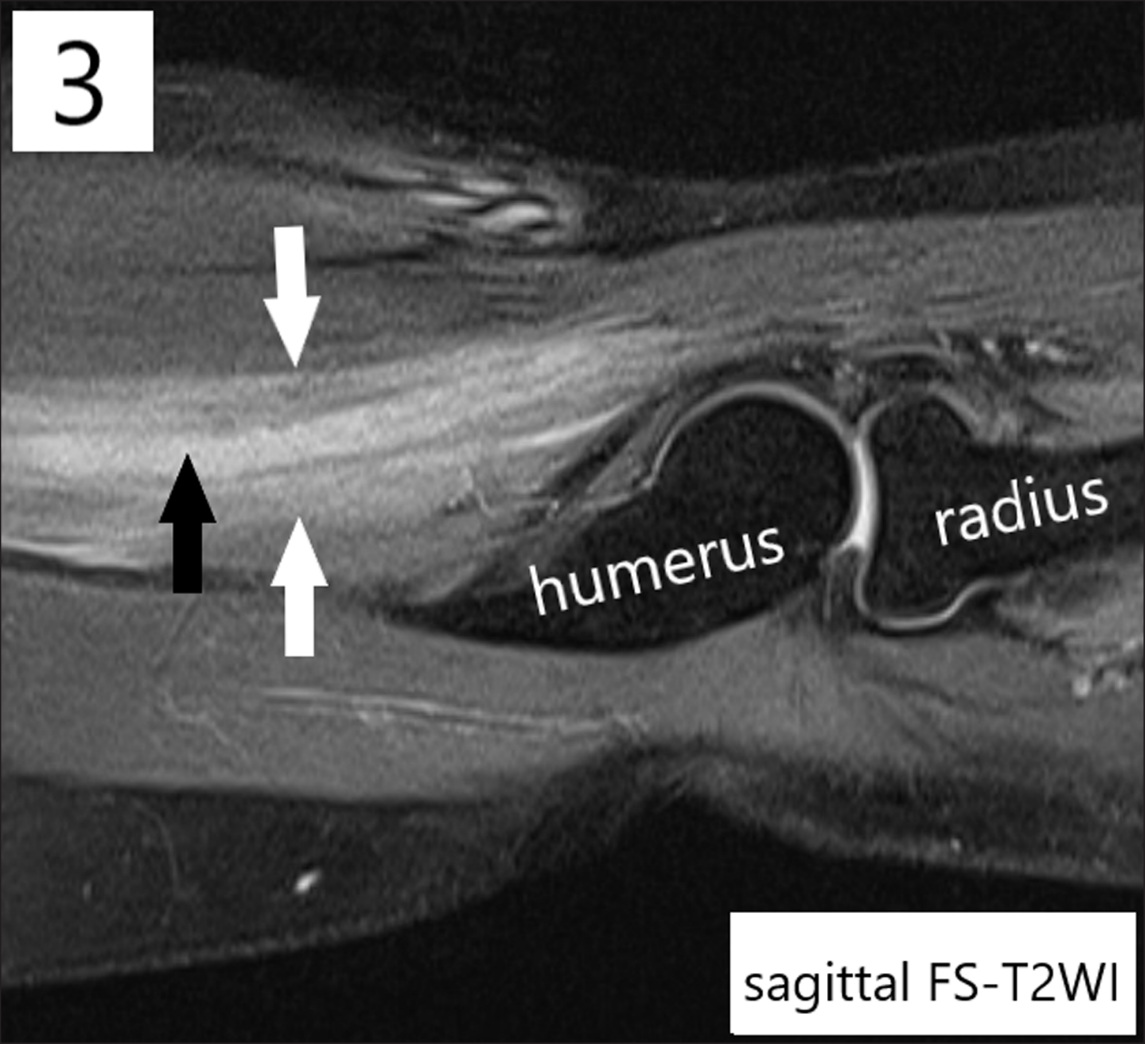


A 32-year-old woman presented with right elbow pain accompanied by loss of strength and sensory disturbances in the forearm. The medical history mentioned a bone marrow transplantation one year prior for acute myeloid leukemia (AML), without current therapy due to assumed remission. Clinical examination revealed hypoesthesia dorsally at digits 1–3, paresis of the hand dorsiflexors, and a pressure-sensitive zone in the upper arm. An electromyogram signalized mononeuropathy of the right radial nerve. Radiographs were negative for osseous/articular elbow lesions. Ultrasound demonstrated a thickened hyporeflective radial nerve in the distal upper arm proximally to the elbow joint. MRI confirmed radial nerve thickening (***Figures 1, 2, 3***, black arrows), measuring 7 cm in craniocaudal diameter and with a maximum axial diameter of 0.7 cm. This pathological nerve tissue was hyperintense on fat-suppressed T2-weighted images (FS-T2WI), isointense to skeletal muscle on T1-weighted images (T1WI), and exhibited avid contrast-enhancement (T1Gd). A triangular perineural zone with similar signal characteristics (***Figures 1, 2, 3***, white arrows) infiltrated the surrounding brachial muscle (*) and brachioradialis muscle (#). Ultrasound-guided biopsy confirmed the presence of an extramedullary acute myeloid leukemia tumor (eAML). A consecutively performed bone marrow biopsy confirmed relapse of the underlying intramedullary/systemic leukemia.

## Comment

An eAML presents with extramedullary masses comprised of immature myeloid cells [1]. An eAML occurs in context of intramedullary/systemic AML, but may also occur as isolated disease without associated pathologic leukemic bone marrow infiltration. The incidence of eAML is underestimated and occurs in 2–8% of newly-diagnosed AML cases. A higher incidence (±15%) occurs in case of relapse of AML after bone marrow transplantation, with a median post-transplant interval to relapse of 6–12 months. Common sites of eAML include the connective tissues (31–35%) and skin (11–46%), with the gastrointestinal system (10–19%), reproductive organs (1–10%), bone (5–16%), head and neck (6–14%), and brain (4–11%) being less frequently involved sites. Magnetic resonance imaging (MRI) typically shows a lesion with homogeneous enhancement that is isointense and hyperintense compared to skeletal muscle respectively on T1- and T2-weighted images. Core biopsy is preferred over fine needle aspiration for histopathological evaluation of eAML as it provides a more reliable assessment of tissue architecture. Bone marrow evaluation is recommended in order to exclude underlying systemic disease. The treatment of eAML consists of leukemia-directed systemic therapy and/or local therapy (e.g., surgery and radiation therapy).
